# Overexpression of the Auxin Binding PROTEIN1 Modulates PIN-Dependent Auxin Transport in Tobacco Cells

**DOI:** 10.1371/journal.pone.0070050

**Published:** 2013-07-23

**Authors:** Milada Čovanová, Michael Sauer, Jan Rychtář, Jiří Friml, Jan Petrášek, Eva Zažímalová

**Affiliations:** 1 Laboratory of Hormonal Regulations in Plants, Institute of Experimental Botany of the Academy of Sciences of the Czech Republic, Prague, Czech Republic, Czech Republic; 2 Department of Plant Systems Biology, VIB (Vlaams Instituut voor Biotechnologie), Ghent, Belgium; 3 Departamento Genetica Molecular de Plantas, Centro Nacional de Biotecnología, CSIC (Consejo Superior de Investigaciones Cientificas), Madrid, Spain; 4 Department of Mathematics and Statistics, the University of North Carolina at Greensboro, Greensboro, North Carolina, United States of America; 5 Institute of Science and Technology Austria (IST Austria), Klosterneuburg, Austria; 6 Department of Functional Genomics and Proteomics, Central European Institute of Technology (CEITEC), Masaryk University, Brno, Czech Republic, Czech Republic; Umeå Plant Science Centre, Sweden

## Abstract

**Background:**

Auxin binding protein 1 (ABP1) is a putative auxin receptor and its function is indispensable for plant growth and development. ABP1 has been shown to be involved in auxin-dependent regulation of cell division and expansion, in plasma-membrane-related processes such as changes in transmembrane potential, and in the regulation of clathrin-dependent endocytosis. However, the ABP1-regulated downstream pathway remains elusive.

**Methodology/Principal Findings:**

Using auxin transport assays and quantitative analysis of cellular morphology we show that ABP1 regulates auxin efflux from tobacco BY-2 cells. The overexpression of ABP1can counterbalance increased auxin efflux and auxin starvation phenotypes caused by the overexpression of PIN auxin efflux carrier. Relevant mechanism involves the ABP1-controlled vesicle trafficking processes, including positive regulation of endocytosis of PIN auxin efflux carriers, as indicated by fluorescence recovery after photobleaching (FRAP) and pharmacological manipulations.

**Conclusions/Significance:**

The findings indicate the involvement of ABP1 in control of rate of auxin transport across plasma membrane emphasizing the role of ABP1 in regulation of PIN activity at the plasma membrane, and highlighting the relevance of ABP1 for the formation of developmentally important, PIN-dependent auxin gradients.

## Introduction

In search for an auxin receptor, an ‘auxin binding site I’ was first identified in 1970s [1,2] and the corresponding ABP1 was later purified from membrane fractions from maize coleoptiles and characterized [3,4]. Maize ABP1 is a small, soluble glycoprotein with N-terminal signal peptide for entry into the secretory pathway and a C-terminal KDEL sequence for luminal endoplasmic reticulum (ER) retention [5,6]. Indeed, ABP1 has been found predominantly in the ER, and only a small part of its population is expected to escape through the secretory system to the outer face of the plasma membrane (PM) [7-10]. Because of the sharp pH optimum at 5.5 for binding of auxin to ABP1 [3], it is predominantly the apoplast/PM-residing fraction of ABP1 that is expected to act as an auxin receptor. At the level of plant organs, ABP1 is expressed primarily in meristems. However, it can be found throughout the whole plant body of all land plants tested [11], and recently ABP1 was localized in tobacco in ovary, egg cells and in embryos at all developmental stages [12,13].

So far no particular protein has been proven *in vivo* to cooperate directly with ABP1 on PM; nevertheless, two candidate proteins were identified in maize using photoaffinity crosslinking with synthetic peptides corresponding to the C-terminus of ABP1 and subsequent mass spectrometry analysis. One of them was GPI-anchored protein homologous to members from SKU5-Similar (SKS) family and the other one belonged to ricin-type lectin family with only one homologous gene in *Arabidopsis* [14].

Early studies suggested that ABP1 mediates rapid ‘non-transcriptional’ responses to auxin occurring on the PM. Antibodies raised against maize ABP1 completely inhibited the electrical response (a shift of transmembrane potential) to auxin in tobacco protoplasts [15]. Together with studies using auxin agonist antibodies [16], these findings suggested that the signal for activation of the relevant H^+^-ATPase is initiated from the cell exterior [17,18]. It has been shown that the auxin-regulated activation of H^+^-ATPase through phosphorylation of the penultimate threonine is indeed independent on TIR1/AFB-dependent auxin signalling, thus supporting the involvement of ABP1 [19]. Furthermore in rice direct interaction between ABP^57^ and H^+^-ATPase has been proven *in vitro* [20]. It has been suggested that the C-terminus of ABP1 is likely to convey the signal for auxin-induced H^+^ extrusion by H^+^-ATPase into the cell wall, and that the concurrent K^+^ influx is followed by water uptake and turgor-driven cell expansion [21].

In tobacco BY-2 cells, reducing ABP1 activity by immuno-modulation resulted in cell-cycle arrest [22]. Constitutive overexpression of *Arabidopsis thaliana* ABP1 (*At*ABP1) in maize cell lines led to the production of larger cells [23]. Inducible overproduction of *At*ABP1 in tobacco plants resulted in larger leaf cells but in no change in leaf size, indicating that the increased cell size was accompanied by reduced cell division. In *Arabidopsis* , a loss-of-function mutation of ABP1 resulted in disoriented cell division and increased cell elongation and resulted in embryo lethality, demonstrating that ABP1 function is indispensable for plant development [24]. Interestingly, reducing the ABP1 activity seems to have opposite effects in shoot and root apices. In the shoot, leaf growth is reduced due to impaired cell expansion so that the cells end up being much smaller [25], whereas a lack of ABP1 in the root prompts the elongation of meristematic cells which are resistant to indole-3-acetic acid (IAA) [26]. Furthermore, the ABP1 activity has been shown to act upstream of Rho GTPase signalling, which mediates the lobed growth of epidermal pavement cells in *Arabidopsis* leaves [27].

The processes of cell expansion and cell division depend on distinct and finely tuned levels of intracellular auxin. The auxin levels are largely regulated by the PM-residing auxin transporters, in particular PIN efflux carriers [28]. Membrane vesicles carrying PIN proteins undergo dynamic recycling to and from the PM [29,30] and auxin regulates its own transport by inhibiting the endocytic step of this recycling [31]. It has been shown that the effect of auxin on endocytosis involves a non-transcriptional mechanism and that ABP1 mediates this effect [32]. Here we concentrated on the mechanism of ABP1 effect(s) on transmembrane auxin transport, namely on the functional consequences of the ABP1-mediated regulation of PIN-dependent auxin efflux. Our results show that in suspension-cultured tobacco BY-2 cells, the overexpression of ABP1 balances the auxin efflux, and that ABP1 acts through regulating the amount and dynamics of PIN auxin efflux carriers at the PM.

## Results and Discussion

To investigate the involvement of ABP1 in the regulation of auxin transport at the cellular level we used tobacco BY-2 cells [33], an established model system for quantitative assays of auxin transport [34]. We generated BY-2 cell lines expressing either *Arabidopsis* *ABP1* under the glucocorticoid (dexamethasone, DEX)-inducible promoter [35] (GVG-*At*ABP1 cells) or tobacco *ABP1* under the constitutive CAMV 35S promoter (35S-*Nt*ABP1 cells). *Arabidopsis* and tobacco *ABP1* genes were also transformed into BY-2 lines expressing *Arabidopsis* *PIN7* (GVG-PIN7 cells [28]) and *PIN1::PIN1: GFP* [36] (PIN1-GFP cells [37]), yielding GVG-PIN7/*Nt*ABP1 and PIN1-GFP/GVG-*At*ABP1 cell lines, respectively. These cell lines allowed us to study simultaneously the effect of ABP1 overexpression on *At*PIN7-dependent auxin transport and the role of ABP1 in *At*PIN1 localization and dynamics.

**Figure 1:**
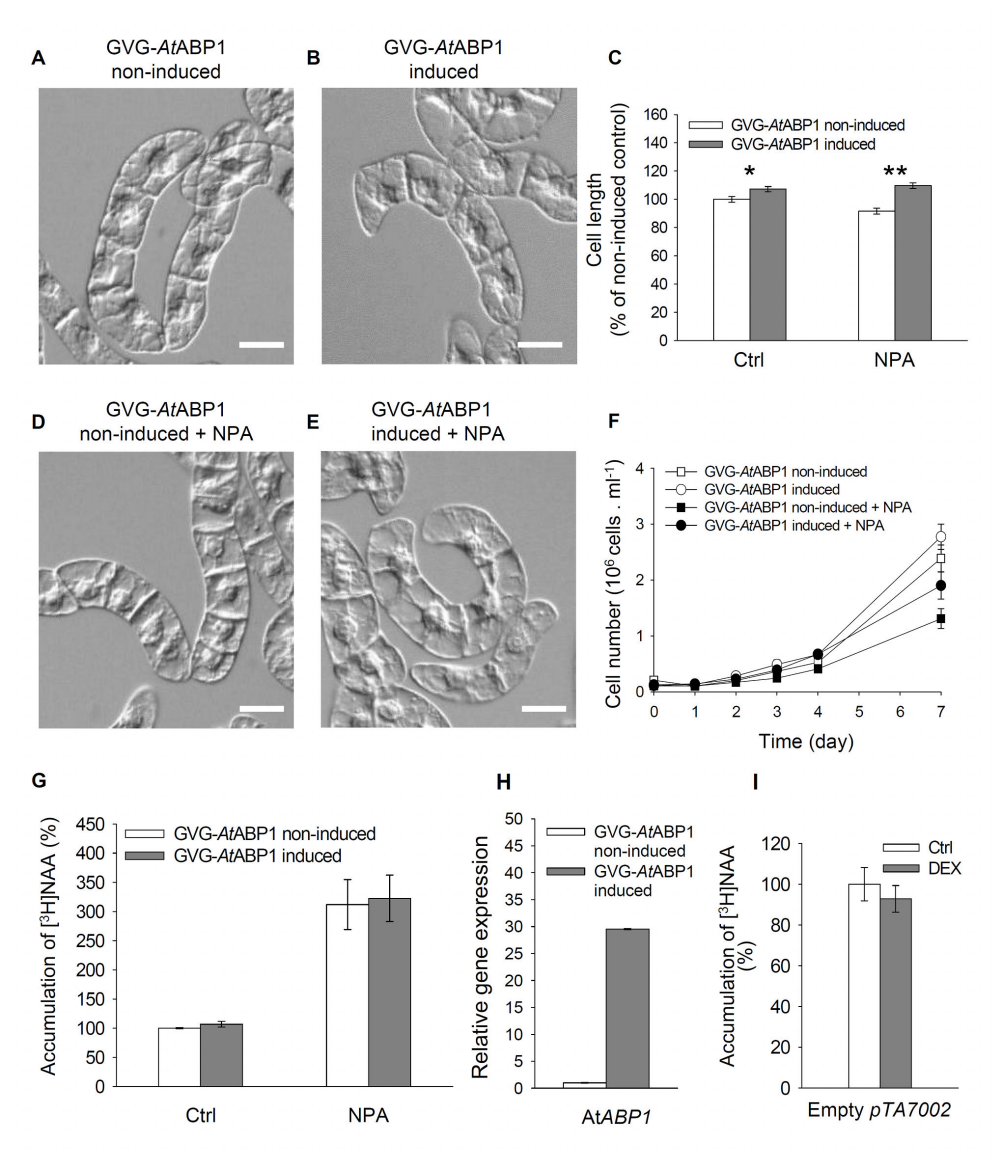
The effects of inducible expression of *At*ABP1 in GVG-*At*ABP1 tobacco BY-2 cells, and treatment with the inhibitor of auxin efflux NPA. (A–E) Morphology of three-day-old non-induced and induced cells, control and NPA-treated (10µM for three days). (A,B,D,E) Nomarski DIC images. Scale bars, 40 µm. (C) Cell length of non-treated (Ctrl) and NPA-treated (10 µM for three days) cells. 100%, value for non-induced control. Error bars, SEM, n~300. Asterisks indicate significantly different means between cells non-expressing and expressing the At*ABP1* gene, two sample t-test assuming unequal variances; *P < 0.005, degrees of freedom (df) = 581; **P < 0.001, df = 573). (F) Growth curves for non-induced and induced cells, non-treated and treated with NPA (10 µM for three days). Error bars, SEM, n=4. (G) Accumulation of [^3^H] NAA as an indicator of the auxin efflux. One-day-old GVG-*At*ABP1 cells were treated with [^3^H] NAA (2 nM) alone (Ctrl) or in combination with NPA (10 µM), and radioactivity was measured after 25 min. Data values are percentages of non-induced, non-treated control (100%). Error bars, SEM, n=3. The differences in [^3^H] NAA accumulation between non-induced and induced GVG-*At*ABP1 cells either without or after NPA application are not statistically significant (P = 0.707 and P = 0.328, respectively, paired samples t-test). (H) Relative expression of the At*ABP1* gene in the GVG-*At*ABP1 cell line. qRT-PCR at 24 hours after induction with dexamethasone (DEX, 1 µM). Error bars, SEM, n=6. (I) Accumulation of [^3^H] NAA in BY-2 cells transformed with an empty pTA7002 vector, measured 25 min after the addition of [^3^H] NAA (2 nM). Data are expressed as percentages of non-treated control (100%), and represent the mean of four technical repetitions. Error bars, SEM, n=4.

**Figure 2:**
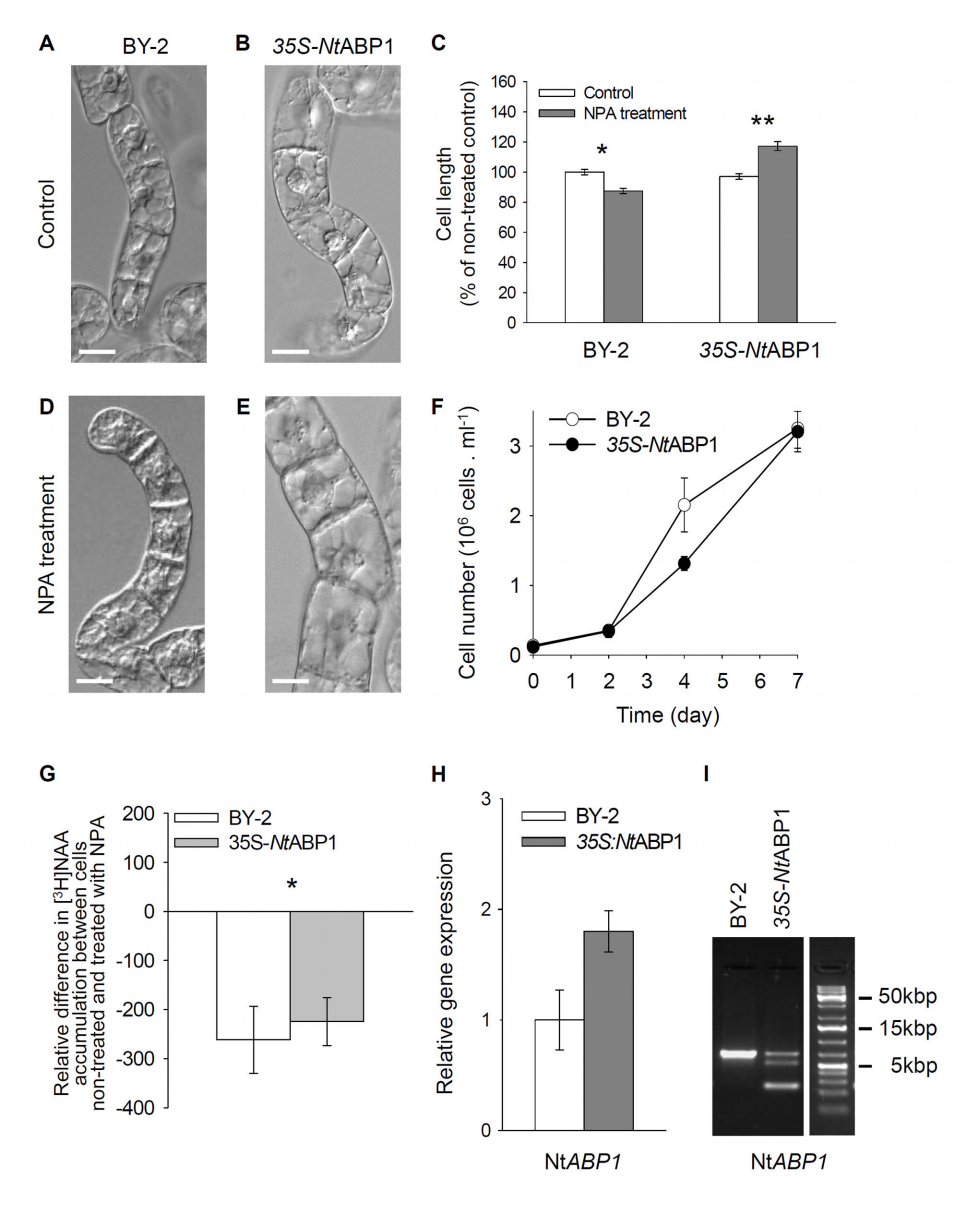
The effects of 35S-driven expression of *Nt*ABP1 in 35S-*Nt*ABP1 tobacco BY-2 cells, and treatment with the inhibitor of auxin efflux NPA. (A–E) Morphology of three-day-old non-induced and induced cells, control and NPA-treated (10µM for three days). (A,B,D,E) Nomarski DIC images. Scale bars, 20 µm. (C) Cell length of non-treated and NPA-treated (10 µM for three days) cells. 100%, value for non-treated BY-2 cells. Error bars, SEM, n~300. Asterisks indicate significantly different means between cells non-expressing and expressing 35S-driven Nt*ABP1* gene. Two sample t-test assuming unequal variances; *P < 0.001, df = 465; **P < 0.001, df = 320. (F) Growth curves for BY-2 and 35S-*Nt*ABP1cells. Error bars, SEM, n=4. (G) Accumulation of [^3^H] NAA as an indicator of the auxin efflux. Difference in [^3^H] NAA accumulation between cells non-treated and treated with NPA is shown for one-day-old BY-2 (control) and 35S-*Nt*ABP1 cells. Radioactivity was measured 25 min after addition of radioactively labelled auxin (2 nM) without or together with NPA (10 µM). Error bars, SEM, n=3. The asterisk denotes statistical significance of difference (P = 0.018 in paired samples t-test). (H,I) Relative expression of Nt*ABP1* gene in control BY-2 and 35S-*Nt*ABP1 cell lines. (H) qRT-PCR from cDNA 24 hours after inoculation of cells into the fresh medium. Error bars, SEM, n=6. (I) PCR of Nt*ABP1* using genomic DNA (702bp fragment of endogenous gene, 256bp fragment of transgenic cDNA).

Phenotypes of DEX-induced GVG-*At*ABP1 cells (Figure 1) and 35S-*Nt*ABP1 cells (Figure 2) were similar to those in the control cell lines, and consisted of cell chains during the exponential growth phase, which gradually disintegrated in the stationary phase. Growth rates (reflecting the cell division activity) in induced GVG-*At*ABP1 cells were similar to those in non-induced controls (Figure 1F); likewise, the growth rates in the constitutively expressing 35S-*Nt*ABP1 cells were similar to those in the corresponding controls (Figure 2F). Nevertheless, when compared with non-induced controls, in three-day-old induced GVG-*At*ABP1 cell line cells were significantly larger (Figure 1C). The extent of cellular phenotypic responses presumably reflects the fact that the DEX induction increased At*ABP1* expression ~30-times (Figure 1H) whereas the 35S-driven expression of Nt*ABP1* was increased only ~two-times (Figure 2H). Positive effect of ABP1 on elongation growth is in agreement with previously reported ABP1-mediated elongation of epidermal cells in tobacco leaves [23,38].

The growth rate and morphology (elongation) of cell files in tobacco cell lines have been reported to be regulated by 1-naphthylphthalamic acid (NPA)-sensitive directional transport of auxin [39-42]. To test whether ABP1 action on cell elongation and division is mediated by auxin efflux, we applied 10 µM NPA to GVG-*At*ABP1 induced and non-induced cells and also to control BY-2 and 35S-*Nt*ABP1 cells at the time 0 (i.e. at the time of cell culture inoculation). After three days of cultivation, a higher proportion of cell files with elongated cells were observed in both induced GVG-*At*ABP1 (Figure 1C, E) and 35S-*Nt*ABP1 lines treated with NPA (Figure 2C, E). Moreover, at the end of the subculture period (day 7), induced GVG-*At*ABP1 cells showed less inhibition of cell division after NPA treatment. NPA treatment reduced cell number by one third compared with reduction by ca. one half in the non-induced GVG-*At*ABP1 cells (Figure 1F). In concert with our results, impaired cell division activity was also observed after the application of a comparable concentration of NPA (12µM) in four-day-old BY-2 cells [43].

To test the effect of constitutive and inducible expression of ABP1 on the NPA-sensitive auxin efflux, we measured the accumulation of radioactively labelled auxin. For this purpose we used the synthetic auxin naphthalene-1-acetic acid (NAA), accumulation of which in tobacco cells reflects mainly the activity of auxin efflux carriers [44]. All auxin efflux assays were performed with one-day-old cells in order to detect only the early ABP1-mediated effects on auxin efflux. [^3^H] NAA accumulation and the effect of NPA were the same in both non-induced and induced GVG-*At*ABP1 cells (Figure 1G). In 35S-*Nt*ABP1 cells the sensitivity of [^3^H] NAA accumulation towards NPA was slightly decreased (Figure 2G). This difference in NPA sensitivity between the inducible GVG-*At*ABP1 line and the constitutive, 35S-*Nt*ABP1 line presumably reflects mechanisms that compensate for long-term stable overexpression of ABP1.

Altogether, both cell phenotype analyses and auxin transport assays suggest a link between ABP1 action, cell division and elongation, and NPA-sensitive auxin efflux. The latter is consistent with a finding that basipetal auxin transport was reduced in heterozygous *abp1/ABP1* mutant [45]. Certainly, the data may reflect downstream changes triggered by the ABP1 at both transcriptional and non-transcriptional levels [46].

**Figure 3:**
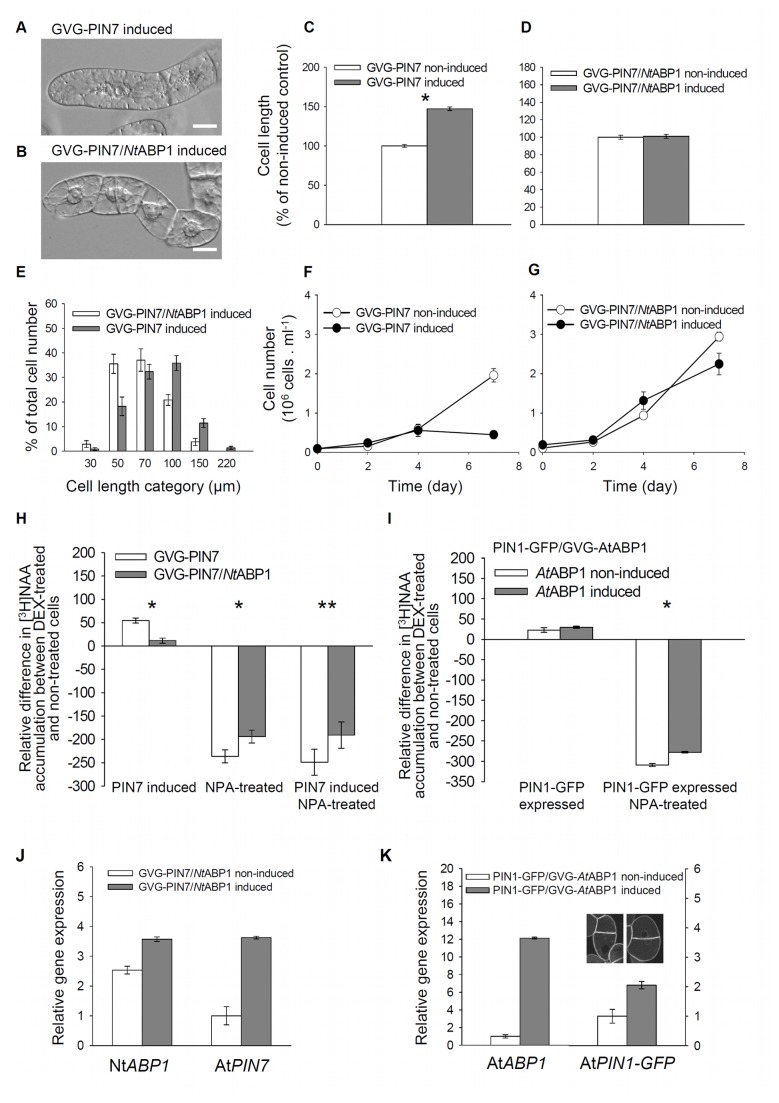
ABP1 prevents tobacco cells from PIN-dependent auxin starvation phenotype, and from excessive auxin efflux. (A–D) Morphology of three-day-old GVG-PIN7 and GVG-PIN7/*Nt*ABP1. (A,B) Nomarski DIC images. Scale bars, 20 µm. (C, D) Cell length of non-induced and induced cells. 100%, value for non-induced cells. Error bars, SEM, n~250. Asterisk indicates significance using two sample t-test assuming unequal variances; (C) *P < 0.001, df = 347; (D) the difference is not statistically significant, P = 0.736, df = 444. (E) Categorized cell-length distribution and (F,G) growth curves of non-induced and induced GVG-PIN7 and GVG-PIN7/*Nt*ABP1 cells. Error bars, SEM, n=10 in E and n = 4 in F, G. (H,I) Accumulation of [^3^H] NAA as an indicator of the auxin efflux, measured 25 min after the addition of [^3^H] NAA to one-day-old cells treated with NPA (10 µM, applied immediately after the addition of [^3^H] NAA, 2 nM). Data are shown as differences in [^3^H] NAA accumulation between induced and non-induced cells (zero level = non-induced/non-treated line). (H) GVG-PIN7 and GVG-PIN7/*Nt*ABP1 cells. Error bars, SEM, n=3. Asterisks indicate significantly different means between cells expressing only the endogenous Nt*ABP1*(GVG-PIN7) and over-expressing the Nt*ABP1* gene (GVG-PIN7/*Nt*ABP1). Paired samples t-test, *P < 0.001, **P = 0.006. (I) PIN1-GFP/GVG-*At*ABP1 cells. Error bars, SEM, n=2. The asterisk indicates significant difference between induced and non-induced NPA-treated cells, P = 0.013, paired samples t-test. There was no significant difference between cell lines without NPA treatment. (J, K) qRT-PCR of Nt*ABP1* and At*PIN7* genes in the GVG-PIN7/*Nt*ABP1 cell line (J), and *AtABP1* and *PIN1-GFP* genes in PIN1-GFP/GVG-*At*ABP1 cell line (K). Expression verified at 24 hours after application of 1 µM DEX. Error bars, SEM, n=6. Inset in (K) shows PIN1-GFP fluorescence in both non-induced and induced cells.

Next, we tested the involvement of ABP1 in the regulation of cellular auxin efflux specifically with respect to the activity and localization of canonical, PM-localized PIN proteins, which are rate-limiting components of auxin efflux [28]. We used double-transformed lines GVG-PIN7/*Nt*ABP1 and PIN1-GFP/GVG-*At*ABP1. Following induction of *AtPIN7* expression by DEX, GVG-PIN7 cells characteristically showed marked elongation ( [28,47]; see also Figure 3A, C, E) and cessation of cell division (Figure 3F). Both responses represent symptoms of auxin starvation in auxin-dependent cell populations [48,49]. In contrast, induction of *PIN7* expression with concomitant 35S-driven *ABP1* expression in GVG-PIN7/*Nt*ABP1 cells neither promoted cell elongation (Figure 3B, D, E) nor reduced cell division activity (Figure 3G). This indicates that the *At*PIN7-mediated auxin efflux is negatively affected by 35S-driven *ABP1* expression. To confirm this possibility and to estimate the activity of auxin efflux carriers, we measured [^3^H] NAA accumulation in these cell lines. As for previous experiments, all auxin efflux assays with *PIN7*-expressing cells were performed with one-day-old cells in order to detect only the early effects on auxin transport. Whereas the 24-h-DEX-induced expression of *AtPIN7* alone in the GVG-PIN7 cell line promoted auxin efflux significantly, the PIN7-dependent stimulation of auxin efflux in the induced GVG-PIN7/*Nt*ABP1 cell line was dramatically reduced (Figure 3H). In the case of 35S-driven Nt*ABP1* expression, the susceptibility to NPA was significantly decreased regardless of the *AtPIN7* expression (cf. the differences between open and grey bars in Figure 3H). Again, this suggests that ABP1 affects predominantly the NPA-sensitive auxin efflux. The complementary approach using PIN1-GFP/GVG-*At*ABP1 cells, where ABP1 expression could be induced on the background of stably expressed PIN1-GFP, also showed lower sensitivity of auxin efflux to NPA after the induction of *At*ABP1 (Figure 3I).

In summary, the auxin accumulation assays on tobacco BY-2 cells presented here provide quantitative data showing that ABP1 negatively regulates PIN-dependent auxin efflux, thus complementing the previous results [32]. In our experimental model, the ABP1 effect on auxin efflux becomes very obvious only after overexpression of PM-localized PIN transporters; this suggests either that there are limitations in the model itself or that ABP1 displays its action under conditions of massive transmembrane auxin flow. The ABP1-related reduction of PIN-dependent and NPA-sensitive auxin efflux indicates that ABP1 either modulates the amount of auxin efflux carriers at the PM (as less auxin efflux carriers would represent fewer target sites for NPA action) and/or that it interferes with a hypothetical NPA-interacting component [50,51] in a pathway regulating PIN activity.

**Figure 4:**
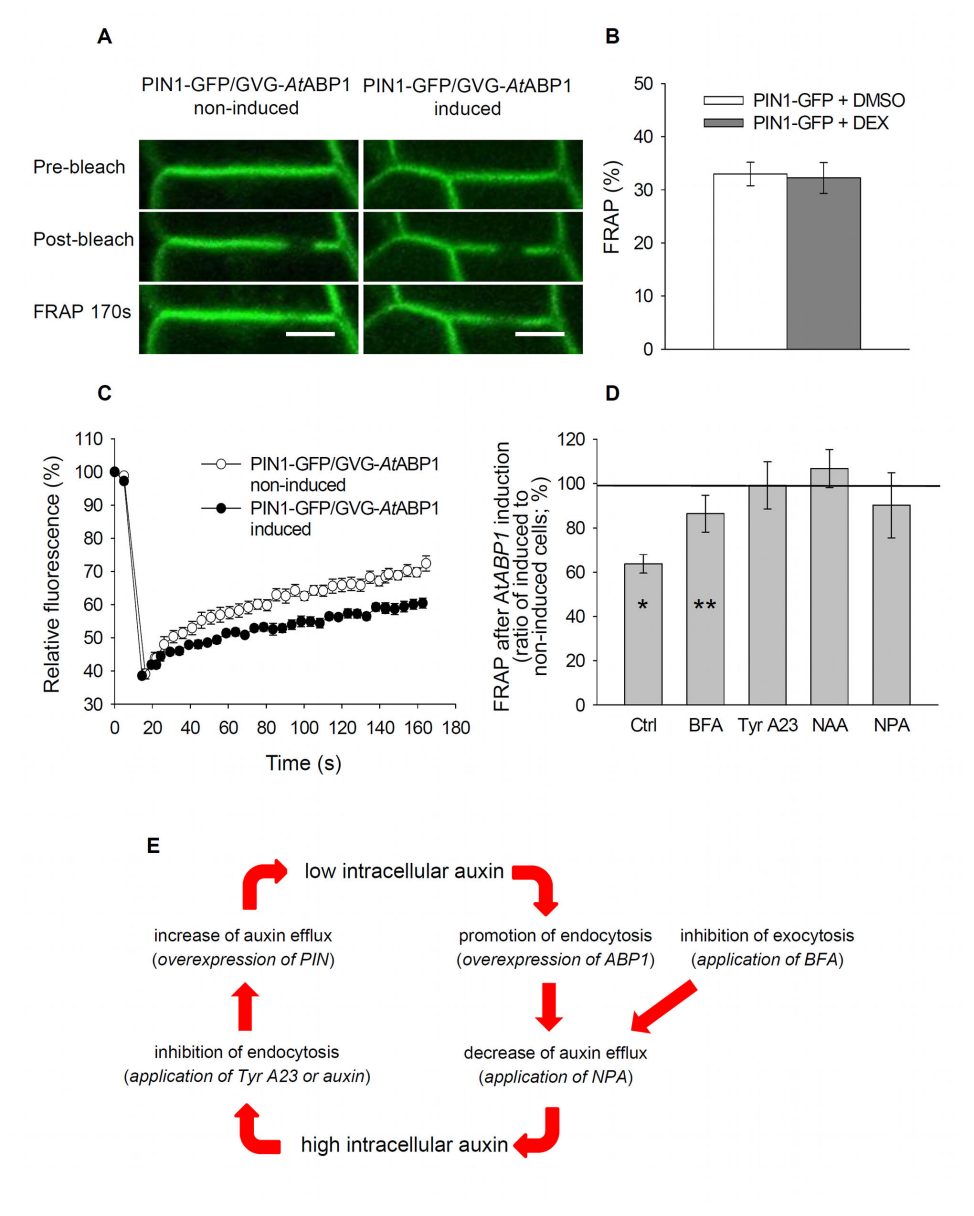
ABP1 inhibits fluorescence recovery after photobleaching (FRAP) of PIN1-GFP. (A–D) FRAP in the three-day-old tobacco PIN1-GFP/GVG-*At*ABP1 and PIN1-GFP cells. (A) Transversal plasma membranes decorated by PIN1-GFP in the PIN1-GFP/GVG-*At*ABP1 cells showing the situation before, immediately after and 170s after the photobleaching. Scale bars, 10 µm. (B) FRAP measured in the PIN1-GFP control cells 170 s after photobleaching. Cells were treated with DEX (1µM) in DMSO or DMSO only. Error bars, SEM, n=15. (C) Kinetics of FRAP in non-induced and induced PIN1-GFP/GVG-*At*ABP1 cells. Error bars, SEM, n=6. (D) Comparison of FRAP after 170 s in cells pre-treated with BFA (20 µM for 30 min), tyrphostin A23 (Tyr A23, 50 µM for 30 min), NAA (5 µM for 60 min), or NPA (10 µM for 25 min). Error bars, SD, Ctrl, n=6; BFA, n=6; Tyr A23, n=6; NAA, n=10; NPA, n=7. FRAP for the PIN1-GFP/GVG-*At*ABP1 non-induced cells, 100%. Asterisks indicate significant difference between *At*ABP1 non-expressing (non-induced) and expressing (induced) cells within given treatment. *P < 0.001, **P = 0.080 using independent samples t-test. The differences for Tyr A23 (P = 0.800), NAA (P = 0.412), and NPA (P = 0.332) treatments are not statistically significant. (E) Schematic depiction of a dual action of ABP1 in regulation of PIN dynamics and activity, resulting in control of auxin levels in a cell. In brackets, experimental intervention is presented. Under low intracellular auxin level, e.g. after overexpression of PIN efflux carriers, ABP1 promotes PIN endocytosis to reduce undesirable auxin export. Under high auxin level, e.g. after external addition of auxin or after inhibition of the active auxin efflux by NPA, ABP1 counteracts the endocytosis of PINs and leaves them on the PM thus promoting the active auxin efflux.

To elucidate whether ABP1 regulates the activity of PIN proteins directly or via changes in their incidence at the PM, we investigated the ABP1-mediated dynamics of PIN proteins in stably transformed BY-2 cells in further details, including their clathrin-mediated endocytosis [32]. We used PIN1-GFP/GVG-*At*ABP1 cells for *in-vivo* confocal microscopy observation of PIN1-GFP dynamics after induction of ABP1 expression. Fluorescence recovery after photobleaching (FRAP) of PIN1-GFP located within the PM of the *At*ABP1-expressing tobacco cells was significantly slower compared to that in non-induced cells (Figure 4A, C, D). DEX itself (used for induction of *At*ABP1 expression) had no effect on FRAP of PIN1-GFP (Figure 4B). These results suggest that ABP1 acts on the resident time of PIN at the PM.

In principle, there are three plausible scenarios to explain this effect: ABP1 may influence endocytosis of membrane vesicles [32], or it may control the deposition of vesicles to the PM, or it may act on both processes simultaneously. To discriminate between these possibilities, we used inhibitors of retrograde (endocytosis) and anterograde (including recycling back to the PM) vesicle trafficking, and quantified FRAP after 170s (Figure 3D). After treatment with the inhibitor of anterograde protein trafficking in plants, brefeldin A (BFA), the FRAP of PIN1-GFP remained slower in the induced ABP1-expressing line compared with that in the non-induced line, suggesting that the effect of ABP1 on PIN1-GFP dynamics is independent of anterograde vesicle transport. In contrast, tyrphostin A23, an inhibitor of recruitment of endocytic cargos (including canonical PINs) into clathrin-mediated endocytic pathway [30], completely abolished the effect of ABP1 expression on PIN1-GFP recovery at the PM. Similarly, treating cells with 5µM NAA, which has been shown to inhibit PIN endocytosis as well [31,32], again prevented the ABP1-mediated decrease of FRAP and even slightly increased the FRAP rate of PIN1-GFP. So, due to binding of auxin (NAA), ABP1 activity may have been inhibited, resulting in even increased recovery of PIN to the PM. Despite high variability, there was no statistical difference between induced and non-induced lines after NPA application, and NPA, similar to tyrphostin A23, seemed to completely abolish the effect of ABP1 expression on PIN1-GFP recovery at the PM. However, in case of NPA this could be also due to the increased intracellular auxin levels resulting from inhibition of auxin efflux and leading to higher binding of auxin to ABP1. After auxin binding, the rate of the ABP1-mediated endocytosis is reduced and the recovery of PIN1 would be faster. Overall, the FRAP observations indicate that ABP1 does act on the dynamics of PIN proteins and that it is a result of the ABP1 effect on endocytosis; they are also consistent with the complementary notion that auxin-induced inhibition of endocytosis is mediated by ABP1 [32].

It was shown previously, that auxin levels affect also the state of actin cytoskeleton [43] and thus determine PIN dynamics [29]. Increasing the amount of auxin inside the cell is characterized by the formation of fine actin filaments which promotes the deposition of new auxin efflux carriers to the PM and in turn reduces the intracellular amount of auxin. Conversely, actin filaments are bundled in cells that are depleted from auxin [52]. All these findings reflect the complex regulatory network by which auxin controls its own levels in cells.

In summary, combined results from the phenotype analysis, auxin efflux measurements as well as FRAP experiments strongly suggest that ABP1 regulates the auxin efflux from cells, and it performs it via control of PIN carriers’ incidence at the PM. The experiments presented here point at a dual function for ABP1 (Figure 4E). Under low cellular auxin levels, e.g. in cells overexpressing canonical, PM-localized auxin efflux carriers, ABP1 reduces cellular auxin efflux by promoting PIN endocytosis to prevent an excessive auxin outflow. Under high auxin levels (e.g. after treatment with NAA or NPA) ABP1 (after binding auxin) mediates inhibition of endocytosis to stimulate export of auxin from the cell. It should be noted here, that so far it is not clear where is the actual site of ABP1 action – at the cell cortex or at the outer side of the PM? Treatment with NAA may saturate ABP1 on both sides of the PM. Treatments with NPA (resulting in higher internal auxin concentration due to inhibition of auxin efflux) may suggest the action of ABP1 inside cells. Anyway, the auxin-dependent action of ABP1 seems to be connected with higher auxin levels; this is in concert with its affinity constant towards IAA (maize K_A_ ca. 10^7^ M^-1^ [3]). A dual role of ABP1 depending on auxin levels is in agreement with results from *Arabidopsis* [32], where ABP1 acted as a positive regulator of clathrin-mediated endocytosis and its action was inhibited by high (above micromolar) auxin levels. Altogether, besides supporting the role of ABP1 in the regulation of PIN endocytosis, this work elucidates the physiological output of this regulation, namely the ABP1-mediated fine-tuning of PIN-dependent auxin efflux.

## Materials and Methods

### Plant material and gene constructs

The tobacco cell line BY-2 (*Nicotiana tabacum* L., cv. Bright Yellow-2 [33]) was cultivated as described [53]. Tobacco BY-2 cell lines carrying *Arabidopsis thaliana PIN7* gene under DEX-inducible promoter (line GVG-PIN7) and intragenic translational *GFP* fusion with *Arabidopsis thaliana PIN1* gene under native promoter (line PIN1-GFP) were described previously [28,54]. For gene transformation, the modified protocol [55] was used as described in [53]. The 35S-*Nt*ABP1 cell line was generated by transformation with *Nicotiana tabacum* cDNA for the *ABP1* gene driven by CaMV35S promoter in pCP60 binary vector [56]; the construct containing *NtABP1* was kindly provided by Catherine Perrot-Rechenmann (CNRS, Gif sur Yvette, France). The GVG-*At*ABP1 cell line was obtained by transformation of *Arabidopsis thaliana ABP1* gene under DEX-inducible promoter in the binary vector pTA7002 [35]. The *GVG-AtABP1* was cloned by inserting PCR-amplified cDNA of *Arabidopsis* *ABP1* into the pTA7002 vector. The GVG-PIN7 cell line [28] was re-transformed with the *NtABP1* construct to create the GVG-PIN7/*Nt*ABP1 cell line. The PIN1-GFP cell line was re-transformed with *GVG-AtABP1* to create the PIN1-GFP/GVG-*At*ABP1 cell line. The pTA7002 line was obtained by transformation of BY-2 cells with the empty vector pTA7002 [35]. Transformed BY-2 cells were maintained in culture media containing 40 µg ml^-1^ hygromycin (cell lines GVG-PIN7 and GVG-*At*ABP1) or 100 µg ml^-1^ kanamycin (cell lines *Nt*ABP1 and PIN1-GFP) or both (cell lines GVG-PIN7/*Nt*ABP1 and PIN1-GFP/GVG-*At*ABP1), and 300 µg ml^-1^ cefotaxim was added to all lines. Expression of *PIN7* and *ABP1* genes in tobacco BY-2 cells was induced by adding DEX (1 µM) from a 30 mM stock solution in DMSO at the beginning of the subcultivation period. The corresponding amount of solvent (DMSO) was added to control cells.

### PCR and qRT-PCR

Tobacco genomic DNA was isolated using DNeasy Plant Mini Kit (Qiagen). Nt*ABP1* gene fragment in 35S-*Nt*ABP1line was amplified by PCR using *Taq* DNA Polymerase Kit (Fermentas). The combination of forward primer (5’-AAACTATGGGAGGTCCGGTT-3’) and reverse primer (5’-AACAGGGATATGGAAGGTGC-3’) produced a product of 250bp in case of transgene in the form of cDNA and 700bp product for the endogenous Nt*ABP1*.

Total RNA was isolated using SpectrumTM Plant Total RNA Kit (Sigma-Aldrich) from transformed one-day-old BY-2 cells. Isolated RNA was treated with DNAse from DNA-freeTM Kit (Ambion), separated in 1% agarose gel and its concentration measured to determine its proper amount for reverse transcription reaction.

For the 25 µl of reverse transcription reaction, first-strand cDNA synthesis, was used 1 µg RNA, 0.5 µg Oligo (dT)_18_ and 50 units of M-MLV Reverse Transcriptase (H-) (Promega).

First strand cDNA was diluted 50x and qPCR was performed using DyNAmoTM Flash SYBR® Green qPCR Kit (Finnzymes) in a final volume 20 µl according to the manufacturer’s manual. Plastic EU Semi skirted Thin-wall Plates (BIOplastics, Landgraaf, The Netherlands) were applied. LightCycler 480 (Roche) was programmed as follows: after 7 min of initial denaturation at 95°C, 40 cycles of 10 s at 95°C for melting, 15 s at 56°C for annealing and 1 min at 70°C for final extension were performed. Primers in 10 µM final concentration were used as follows: Nt*ABP1* forward 5‘-AAACTATGGGAGGTCCGGTT-3‘, reverse 5‘-AACAGGGATATGGAAGGTGC-3‘; At*ABP1* forward 5‘-TGTGAAGAGGTTTTTGTTGTCC-3‘, reverse 5‘-GCAGCAGTGTGTGGCATAA-3‘; At*PIN7* forward 5‘-GGGAAGAAGAGTCGGAGAG-3‘, 5‘-AAGAGCCCAAATGAGACCAA-3‘; At*PIN1* forward 5‘-GCTGGGAGGTTTCATTATC-3‘, reverse 5‘-GTTTCCGTCTTGTCTTTTC-3‘; Nt*ACT2* forward 5‘-CTATTCTCCGCTTTGGACTTGGC-3‘, reverse 5‘-AGGACCTCAGGACAACGGAAACG-3‘. 

PCR efficiencies were estimated from calibration curves generated from serial dilution of cDNAs. The calibrated normalized ratios of the relative amount of the target and reference gene were calculated as published in Cháb et al. [57]: E_R_^CpR^x E_T_^-CpT^ where E_T_, E_R_ are the efficiencies for target or reference gene qRT-PCR assay, and CpT, CpR a crossing points for target or reference genes. Expression levels were normalized against Nt*ACT2* as the reference gene. Resulting values are expressed as a ratio of relative expression of particular gene in transformed/induced cells against relative expression of this gene in non-transformed/non-induced cells. Every sample was measured in three biological repetitions which were in duplicates giving 6 repetitions in sum.

### Microscopy, image analysis and determinations of cell growth parameters

Nomarski DIC microscopy was performed using Nikon Eclipse E600 microscope (Nikon, Japan), and images were grabbed with colour digital camera (DVC 1310C, USA). Cell length was measured interactively using LUCIA image analysis software (Laboratory Imaging, Prague, Czech Republic) and expressed as a percentage of mean length of control cells. The categorized length distribution of cells from each of 10 optical fields of each sample was presented as the percentage of total cell number (~200 cells). Each category corresponds to the maximum value of each cell-length interval (0-30, 30-50, 50-70, 70-100, 100-150, 150-220 µm). The diameter of the cells did not change during the subcultivation period and varied between 25–40 µm. Cell density was determined by counting the cells using a Fuchs-Rosenthal haemocytometer slide, performed periodically during the entire subcultivation period; individual values represent the average of at least four aliquots of every sample. A stock solution of NPA (100 mM) in DMSO was added to the cultivation medium at the beginning of the subcultivation period to a final concentration of 10 µM.

### Confocal microscopy and FRAP

Zeiss LSM 5 Duo confocal microscope (Carl Zeiss, Jena, Germany) with appropriate filter sets for GFP detection (excitation 488 nm, emission 505-550 nm) and 40x C-Apochromat water immersion objective (NA=1.2) was used. For FRAP experiments, a rectangular region of interest (ROI) of 40x20 pixels with the PM in the centre was applied interactively at the transversal PMs of the cell files. Bleaching with maximal laser intensity was followed by 170 seconds tracking of fluorescence recovery with imaging every 7 seconds. For the compensation of the fluorescence bleaching during recovery period, rectangular ROI (100x20pixels) was applied using Carl Zeiss Image Examiner software on non-bleached part of the transversal PM. The fluorescence in this ROI was measured and FRAP values were corrected for this background. Presented values are displayed as means of at least 6 measured cells (one ROI/cell) expressed as proportion of the initial signal intensity before photobleaching. For all FRAP experiments, three-day-old cells induced by 1 µM DEX in DMSO at the beginning of subcultivation were used. For inhibitor studies, the cells were pre-treated for 30 minutes before the FRAP experiment with 50 µM tyrphostin A23 (Sigma-Aldrich, USA) added from a 50 mM stock solution in DMSO, or alternatively for 60 min with 5 µM NAA (Sigma-Aldrich, USA) added from a 100 mM stock solution in ethanol, or for 25 min with 10µM NPA added from a 100 mM stock solution in DMSO. The corresponding amount of solvent was added into controls.

### Auxin accumulation assays

Auxin accumulation in one-day-old cells was measured as described in Delbarre et al. [44] and Petrášek et al. [53]. We used cells cultivated for 24 h from the beginning of the subcultivation period unless stated otherwise. At the beginning of the accumulation assay, [^3^H] NAA (20 Ci mmol^-1^; American Radiolabeled Chemicals, Inc., St Louis, MO, USA) was added to the equilibrated cell suspension to a final concentration of 2 nM. Measured values were expressed as percentages of controls at 25 min after the addition of the labelled auxin. NPA was added at the beginning of the accumulation assay from 100 mM stock solution in DMSO to a final concentration of 10 µM. Corresponding volumes of DMSO alone were added as negative controls. Cells transformed with empty vectors were checked for auxin accumulation and showed no significant reaction to DEX (Figure 1I).

### Statistical analysis

Data analysis of cell length differences was performed using two sample t-test assuming unequal variances on several independent biological replicates. Data analysis for accumulation assays was performed using SPSS. The One-Sample Kolmogorov-Smirnoff test was used to check data for normality, and The Paired samples t-test was used to test for statistical differences between means. In cases where the data did not show convincingly normal distribution, we used the Related samples Wilcoxon signed rank to confirm the results. Data analysis for FRAP experiments was performed using SPSS and the One-sample Kolmogorov-Smirnoff test to confirm data normality. An Independent samples t-test was used to test for statistical differences between the means.

### Accession Numbers

The Arabidopsis Genome Initiative locus identifiers for genes used in this study are as follows: *ABP1*, At4g02980; *PIN7*, At1g23080; *PIN1*, At1g73590. The National Center for Biotechnology Information accession number for *NtABP1* is P33490.
