# Correction: Overexpression of the Auxin Binding PROTEIN1 Modulates PIN-Dependent Auxin Transport in Tobacco Cells

**DOI:** 10.1371/annotation/2fa354b5-a4e8-45ca-8eec-342aaa03f2e4

**Published:** 2013-08-20

**Authors:** Milada Čovanová, Michael Sauer, Jan Rychtář, Jiří Friml, Jan Petrášek, Eva Zažímalová

There was an error in the capitalization of the protein in the title.

The correct title is: Overexpression of the AUXIN BINDING PROTEIN1 Modulates PIN-Dependent Auxin Transport in Tobacco Cells

The correct citation is: Čovanová M, Sauer M, Rychtář J, Friml J, Petrášek J, et al. (2013) Overexpression of the AUXIN BINDING PROTEIN1 Modulates PIN-Dependent Auxin Transport in Tobacco Cells. PLoS ONE 8(7): e70050. doi:10.1371/journal.pone.0070050 

